# Transcriptomic alterations in host parasitoid wasps resulting in extended lifespan due to PpNSRV-1 infection

**DOI:** 10.1007/s44297-024-00029-w

**Published:** 2024-06-05

**Authors:** Cheng Xue, Fei Wang, Qi Fang, Shijiao Xiong, Gongyin Ye

**Affiliations:** 1 State Key Laboratory of Rice Biology and Breeding, Institute of Insect Sciences, Zhejiang University, Hangzhou, 310058 China; 2 Hainan Institute of Zhejiang University, Sanya, 572025 China; 3 Xianghu Laboratory, Hangzhou, 311231 China

**Keywords:** Virus-host interaction, Lifespan, RNA-seq, WGCNA, Hedgehog pathway

## Abstract

The parasitoid wasp *Pteromalus puparum*, a pivotal agent in agricultural ecosystem balance, experiences significant alterations in lifespan due to infection by the negative-sense single-stranded RNA virus PpNSRV-1. This study utilized RNA sequencing (RNA-seq) to investigate the genetic mechanism(s) driving lifespan extension in response to this virus. Lifespan analysis, combined with RNA-seq of nearly isogenic lines of infected and uninfected wasps, yields a comprehensive temporal expression profile aligned with the *P. puparum* genome. Despite a modest overall impact of PpNSRV-1 on gene expression, notable effects were observed on specific gene families, such as the SPOP genes and the cytochrome P450 family. Through Weighted Gene Co-expression Network Analysis (WGCNA), we identified connections between the green and greenyellow modules to viral infection traits, with further functional analyses highlighting the pivotal roles of the Hedgehog, autophagy, AMPK, mTOR, p53, and PI3K-Akt signaling pathways. RNA interference experiment targeting the SPOP gene *PPU06594-RA* confirmed its role in lifespan modulation, underscoring the importance of Hedgehog pathway in the host’s adaptive response. These insights not only elucidate the complex genetic landscape of host-virus interactions but also open avenues for innovative biological pest control strategies.

**Supplementary Information:**

The online version contains supplementary material available at 10.1007/s44297-024-00029-w.

## Introduction

Parasitoid wasps, belonging to the order Hymenoptera, are characterized by their egg-laying behavior in or on other arthropods, which then serve as hosts for their offspring’s development. Parasitoid wasps are pivotal in biological control of agricultural and forestry pests due to their species diversity and multifaceted ecological roles [1]. With over 100,000 identified species, including more than 30,000 in China, these wasps represent a vast biological resource with significant potential for ecological conservation and application [2].

Viruses are ubiquitous intracellular parasites, inhabiting all life forms on Earth, spanning plants, animals, fungi, and bacteria [3]. The field of virology has advanced significantly, especially with the advent of molecular biology techniques. Recent developments in high-throughput sequencing (HTS) have enabled the discovery of numerous RNA viruses in various invertebrates. The vast genetic diversity of viruses and their intricate host interactions, including host-hopping and co-evolution, are gaining attention. Studies indicate prevalent virus infections in arthropods (e.g., insects), with their viral genetic diversity surpassing prior estimations. Indeed, the genetic variety of arthropod-associated viruses may rival that of viruses in plants and vertebrates [4]. As with other insect species, a number of viruses or virus-like particles (VLPs) are found on parasitoid wasps. The commensal viruses in parasitoid wasps are diverse and include four genotypes from at least 10 families: double-stranded DNA (dsDNA) viruses, positive-sense single-stranded RNA ((+)ssRNA) viruses, negative-sense single-stranded RNA ((-)ssRNA) viruses and segmented double-stranded RNA (dsRNA) viruses [5, 6]. Of these parasitoid wasp-associated viruses, only a small fraction of RNA viruses have been confirmed to be associated with host survival. For example, Rondani’s wasp virus 1 (RoWV-1), which is transmitted by the parasitoid wasp *Pachycrepoideus vindemmiae*, enters *Drosophila melanogaster* during parasitism. RoWV-1 enhances the oviposition capacity and prolongs the developmental duration of *D. melanogaster*, thereby ensuring that the parasitoid wasps have more hosts for the reproduction of offspring [7]. Recently, multiple novel RNA viruses have been identified by high-throughput sequencing in a dozen species of parasitoid wasps, signaling that parasitoid wasps and RNA viruses may be even more closely linked [8–10].

In a previous study, we reported the discovery and characterization of a novel (-)ssRNA virus in *P. puparum*, named Pteromalus puparum negative-sense single-stranded RNA virus 1 (PpNSRV-1) [6]. ICTV has recently established a novel family *Artoviridae*, by combining the monospecific genus *Peropuvirus*, represented by PpNSRV-1, with six novel viruses of invertebrate [11]. *P. puparum* is a parasitoid wasp that uses a broad range of pest species as hosts [12]. Their primary host is *Pieris rapae*. Field surveys document high parasitism on *P. rapae*, with proportions ranging from 59–62% in winter and up to 90% during early summer [13]. The efficacy of pest control is strongly influenced by the fitness of wasps, which includes their survival, lifespan and sex ratio of offspring. PpNSRV-1 infection was detected in eggs, larvae, pupae and adults of both sexes, with highest titers in female adults. Meanwhile, tissue distribution showed that the virus was present in all tissues of both male and female adults, but was significantly enriched in the ovaries of female wasps. PpNSRV-1 is transmitted vertically from infected parents to offspring. The viral load of PpNSRV-1 accumulates rapidly within 4 days after eclosion and peaks at the 5^th^ day. Natural endemic infections were found in 16–37% of field populations in different locations in China. A key finding was that PpNSRV-1 infection significantly extends the lifespan of *P. puparum* wasp adult, and manipulates the secondary sex ratio of *P. puparum* offspring by reducing female proportions. However, the underlying mechanisms of the virus-induced lifespan extension of wasps remain largely unexplored and warrant further study.

In this study, we aimed to investigate the mechanism by which PpNSRV-1 affects the lifespan of its host wasp, *P. puparum*. We conducted a temporal transcriptomic analysis of the wasps infected with PpNSRV-1, aiming to identify the genes and molecular pathways involved in prolonging the wasp’s lifespan. Our findings expand the interplay between viruses and insects, and can potentially inform the development of novel biological control strategies that leverage the natural enemies of insect pests.

## Results

### Lifespan extension confirmation and sequencing overview

**Fig. 1:**
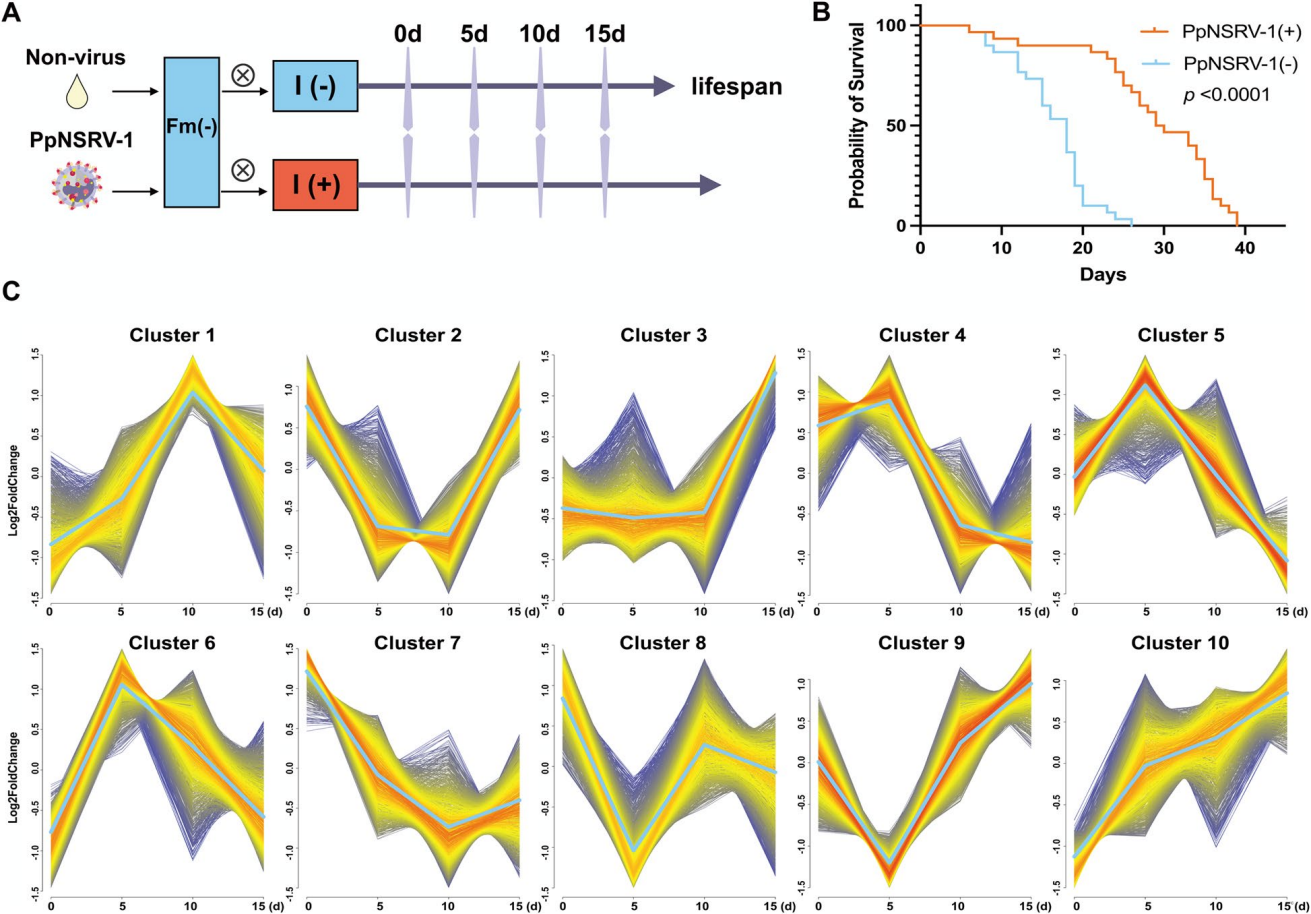
Overview of the construction and transcriptome sequencing of the near-isogenic lineage wasp. **A** The process of constructing the near-isogenic lineage of the *Pteromalus puparum*; **B** Survival rate of female adults of the two lines; **C** Fuzzy c-means clustering identified ten distinct temporal patterns of proteins expression. The x axis represents four developmental stages, while the y axis represents log_2_-transformed, normalized intensity ratios in each stage

Female adult wasps selected for transcriptome sequencing were categorized into I(-) individuals, free from PpNSRV-1 infection, and I(+) individuals, infected with PpNSRV-1 virus, originating from Inject(-) and Inject(+) colonies, respectively (Fig. 1A). We conducted parallel sequencing and lifespan trait validation on both groups, demonstrating that the I(+) wasps exhibited significantly greater survival rate and longer average lifespan compared to I(-) wasps (*p* < 0.0001, Fig. 1B), corroborating earlier findings.

The RNA-seq data from 24 samples, representing both virus-infected and uninfected groups across four different ages, indicating that the percentage of Q30 base exceeding 94%, reflecting high sequencing quality. In total, 803,858,185 high-quality sequencing reads were obtained (Table S1). Following aligning with the reference genome of *P. puparum*, Mfuzz clustering identified genes with similar expression profiles across various age groups, elucidating the expression patterns of 13,194 genes. We observed no consistent trend of sustained gene up- or down-regulation. The clustering analysis revealed a balanced gene distribution, ranging from 1,114 to 1,404 genes per cluster (Fig. 1C). Considering the viral dynamics, especially the progressive accumulation in wasps from day 0 to 5 and the subsequent stabilization of the viral load, our analysis centered on gene clusters that demonstrated significant expression differences between these two critical time points. We highlighted clusters 10, 7, 9, and 5, which showcased distinct expression trends. These four clusters showed the most significant trends in gene expression at the 0–5 d developmental stage. Kyoto Encyclopedia of Genes and Genomes (KEGG) pathway enrichment analysis confirmed the biological significance of these clusters, revealing substantial pathway enrichments (*q* < 0.05) and underscoring their potential roles in the wasp’s response to viral infection. Results of KEGG pathway enrichment analysis revealed significant enrichments within the four clusters (*q* < 0.05), and the remaining clusters showed little to no pathway enrichment. Genes within cluster 5 exhibited a diverse and extensive range of significant enrichment across a multitude of pathways. This cluster demonstrated enrichments in 84 pathways, including the PI3K-Akt signaling pathway, Foxo signaling pathway, MAPK signaling pathway, Autophagy, Longevity regulating pathway, and Apoptosis. Genes within cluster 9 exhibited significant in four pathways, including Coronavirus disease, Parkinson disease, Prion disease and Ribosome (Table S2).

### Differential gene expression patterns in response to PpNSRV-1 infection

**Fig. 2:**
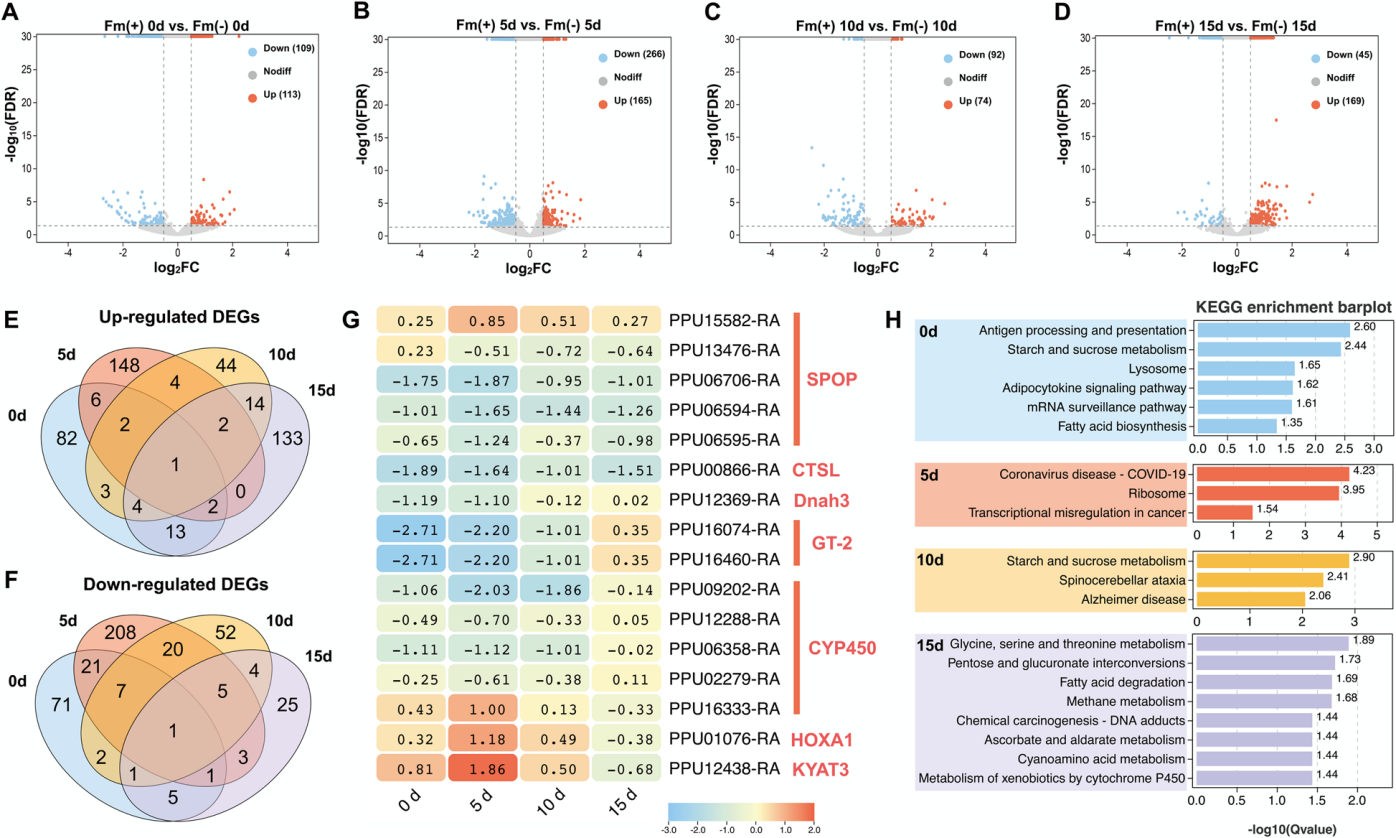
Identification and analysis of differential expressed genes. **A**-**D** Scatter plots of genes at 0–15 d in the I(+) and I(-) lines are shown, where each point represents a gene, with log_2_FC on the x-axis and -log_10_(*p* value) on the y-axis. Genes with log_2_FC > 1 and *p* value < 0.05 were identified as up-regulated genes and marked in orange. Genes with log_2_FC < -1 and *p* value < 0.05 were identified as down-regulated genes and marked in blue. Other genes were labeled as nondiff and marked in gray; **E** Venn diagram of up-regulated DEGs at 4 ages; **F** Venn diagram of down-regulated DEGs at 4 ages; **G** Heatmap of selected DEGs. Each row represented a gene, each column represented an age. Each cell represented the log_2_FC value of a gene; **H** Result of KEGG pathway enrichment. x-axis represented -log_10_(*q* value) of the pathway

Differential expression analysis revealed that PpNSRV-1 induced a relatively modest change on the gene expression profiles in wasps (Fig. 2A-D). Overall, the number of down-regulated genes exceeded that of up-regulated genes. The highest differential gene expression occurred on day 5, coinciding with the peak titer of PpNSRV-1, with 266 genes significantly down-regulated and 165 genes up-regulated. The gene *PPU15685-RA* was consistently up-regulated across all age groups and encodes an unknown protein (Fig. 2E). Conversely, *PPU06594-RA* was significantly down-regulated across all age groups, encoding a Speckle-type pox virus and zinc finger (POZ) protein (SPOP) (Fig. 2F). Notably, several SPOP and Cytochrome P450 family genes (P450s) were identified as differentially expressed genes (DEGs) (Fig. 2G). Specifically, *PPU15582-RA* was up-regulated throughout four ages, peaking at day 5, suggesting a positive role in the initial viral response. *PPU13476-RA* showed down-regulation from day 5 onwards, indicating potential activation of inhibitory regulatory mechanisms in later infection stages. Three SPOPs, *PPU06706-RA*, *PPU06594-RA*, and *PPU06595-RA* were significantly down-regulated from day 0, implicating their potential role in the inhibitory response or cell death pathways post-infection. Cathepsin L (*PPU00866-RA*), a lysosomal protease, exhibited significant down-regulation at three age groups, indicating a potential suppression of lysosome-associated degradation pathways during the early stages of infection. Dynein (*PPU12369-RA*), Trihelix transcription factors (*PPU16074-RA* and *PPU16460-RA*), and P450s, primarily exhibited down-regulation during the early stages of the adult wasps’ lifespan. This pattern suggests significant shifts in cellular functions such as structure integrity, gene expression, and metabolic activities at the onset of viral infection. Homeobox protein Hox-A1 (*PPU01076-RA*) and Kynurenine-oxoglutarate transaminase 3 (*PPU12438-RA*) are usually associated with developmental processes and amino acid metabolic pathways. Their expression was up-regulated at 5 d and may be associated with tissue repair or metabolic adjustments. The expression pattern of these genes may reflect complex biological alterations in parasitoid wasps following viral infection. The KEGG enrichment results of genes in different age groups also emphasized that antiviral, tumor transcriptional misregulation and ribosome-associated pathway genes responded to viral replication at 5 d (Fig. 2H).

### Gene regulatory network in response to PpNSRV-1 virus infection

We applied Weighted Gene Co-expression Network Analysis (WGCNA) to construct a regulatory network and investigate the transcriptional mechanisms contributing to the lifespan extension in I(+) wasps. Principal component analysis (PCA) on gene expression profiles clearly differentiated I(+) from I(-) groups, with one outlier (‘FM-Pp5d-4’) removed for result accuracy before proceeding with WGCNA analysis (Fig. S1).

**Fig. 3:**
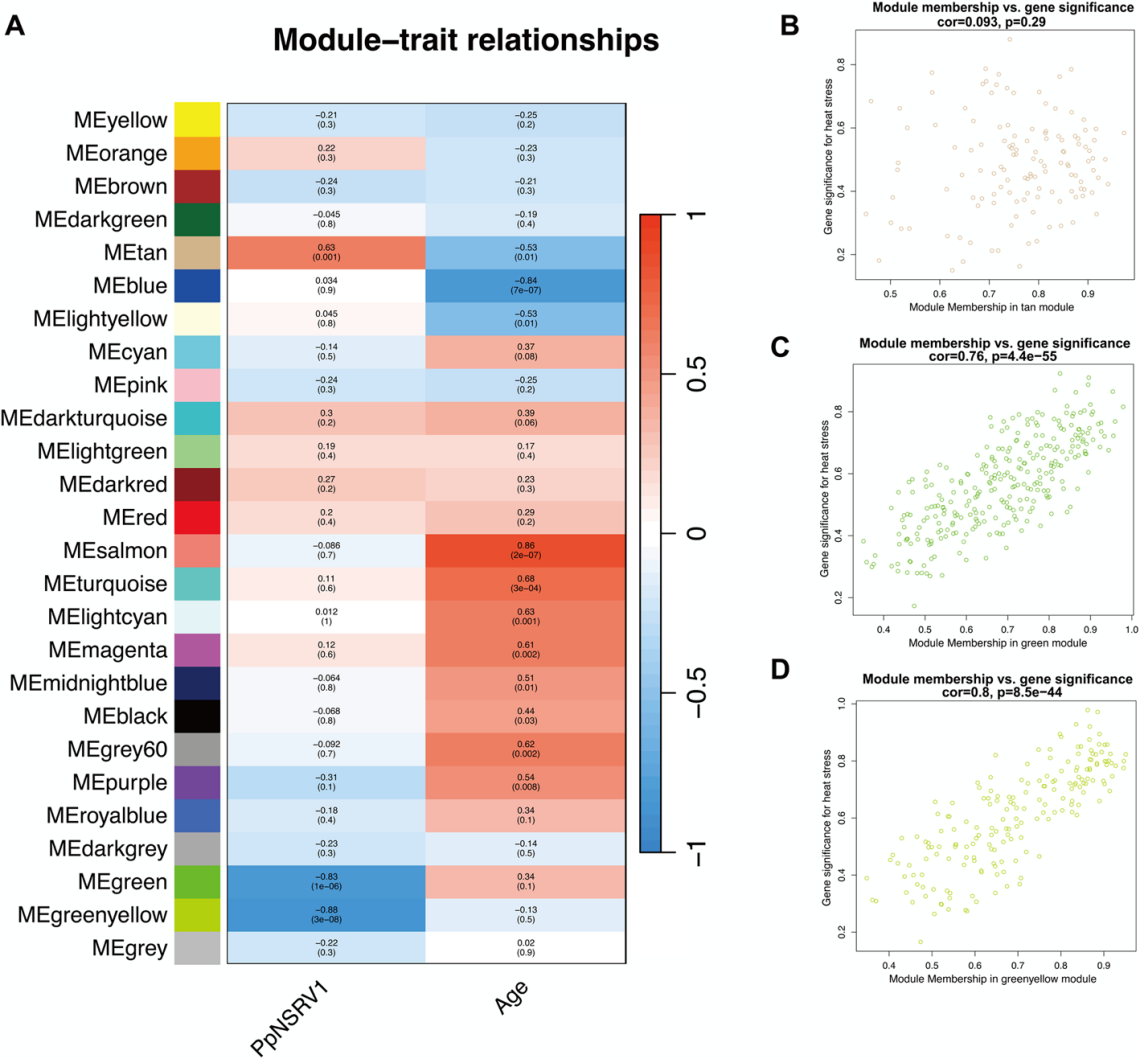
Network constructed via Weighted Gene Co-expression Network Analysis. **A** Heatmap depicting the correlation between modules and phenotypic traits; **B** Scatterplot illustrating the correlation between module membership and gene significance within the tan module; **C** Scatterplot illustrating the correlation between module membership and gene significance within the green module; **D** Scatterplot illustrating the correlation between module membership and gene significance within the greenyellow module

Initially, we determined the optimal soft threshold by balancing network scale and average connectivity within a power parameter range of 1 to 20. The goal was to enhance strong gene correlations within modules, minimize weak inter-module correlations, and maintain essential network topology. A power value of 0.85, corresponding to the minimum scale-free topology fitting index value of 8, was identified as the optimal threshold (Fig. S2A). Subsequent hierarchical clustering of gene expression patterns yielded 26 modules, including unclustered genes in the gray module (Fig. S2B). For each module, the module eigengene (ME) was calculated, serving as a summary of the module’s gene expression profile. Correlation analysis between MEs and transcriptome traits revealed significant correlations for three modules (tan, green, and greenyellow) with PpNSRV-1 infection traits, and ten modules with age (Fig. 3A). Specifically, the tan module positively correlated with PpNSRV-1 infection (*r* = 0.63, *p* = 0.0001) and negatively with age (*r* = -0.53, *p* = 0.01). The green and greenyellow modules showed strong negative correlations with PpNSRV-1 infection (*r* = -0.89 and -0.88; *p*-values of 10^–6^ and 3 × 10^–8^, respectively), indicating gene expression decline post-infection, with no significant correlation to age. The ten age-associated modules may play roles in lifespan regulation, metabolism, or immunity.

Further analysis involved correlating module membership (MM) and gene significance (GS) to assess functional relevance. In the tan module, a weak and nonsignificant positive correlation was observed between MM and GS (*r* = 0.093, *p* = 0.29, Fig. 3B). Conversely, the green and greenyellow modules demonstrated robust correlations between MM and GS (*r* = 0.76 and 0.8; *p*-values of 4.4 × 10^–55^ and 8.5 × 10^–44^, respectively, Fig. 3C-D), suggesting that gene connectivity within these modules and their association with viral infection are highly consistent.

**Fig. 4:**
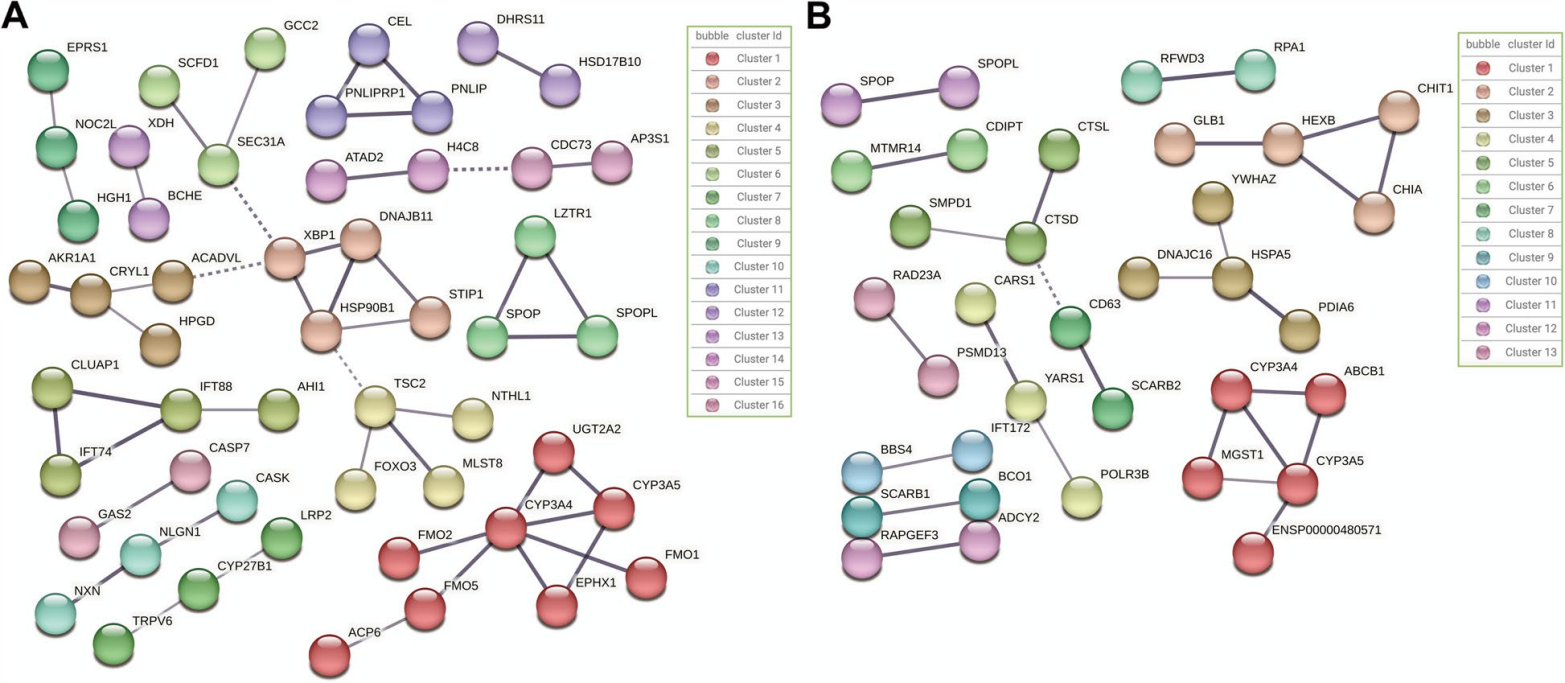
STRING functional network analysis. **A** Functional network of green module genes; **B** Functional network of greenyellow module genes

Therefore, the green module and greenyellow module were selected for further analysis and research. To further investigate the functional roles of genes in the green and greenyellow modules, the protein sequences of all 286 members of the green module were submitted into the Search Tool for the Retrieval of Interacting Genes (STRING) database, using *Homo sapiens* as the reference species for constructing the functional network. After filtering out nodes with low connectivity, the refined network consisted of 120 genes. Given the network’s considerable dispersion, Markov Clustering (MCL) was applied, segmenting 52 interacting members into 16 distinct sub-networks (Fig. 4A). KEGG enrichment analysis of these sub-networks highlighted key biological processes and metabolic pathways.

Cluster 1 featured a variety of P450s (*CYP3A5* and *CYP3A4*) and other enzymes (*FMO2* and *UGT2A2*), predominantly involved in drug metabolism, xenobiotic metabolism, chemogenicity, steroid hormone biosynthesis, retinoid metabolism, and bile secretion pathways (Table 1). These findings suggest these proteins may be pivotal in metabolism, hormone synthesis, and toxin elimination during PpNSRV-1 infection. Meanwhile, Cluster 2 emphasized protein processing in the endoplasmic reticulum with key proteins like XBP1, HSP90B1, and DNAJB11, suggesting involvement in protein folding, quality control, and stress response post-infection. Cluster 3, enriched in pentose and glucuronide conversion pathways with proteins like CRYL1 and AKR1A1, suggested a role in carbohydrate metabolism and detoxification. Importantly, TSC2, FOXO3, and MLST8 in Cluster 4, linked with various pathways including PI3K-Akt signaling, autophagy, mTOR, AMPK signaling, cellular senescence, and thermogenesis, highlighted their critical roles in cell proliferation, growth regulation, nutrient and energy adaptation, and cellular aging. These pathways may contribute to the significant lifespan extension observed in adult host parasitoid wasps post-PpNSRV-1 infection. Additionally, the enrichment of SPOPL and SPOP in the Hedgehog signaling pathway in Cluster 8 underscores their potential importance in cell signaling and development.

**Table 1:** KEGG enrichment for the green module STRING functional network

**Cluster**	**KEGG term**	**Term description**	**Matching proteins in network**	**FDR**
1	hsa00982	Drug metabolism - cytochrome P450	FMO2, CYP3A5, FMO5, FMO1, UGT2A2, CYP3A4	1.51E-11
	hsa00980	Metabolism of xenobiotics by cytochrome P450	CYP3A5, UGT2A2, EPHX1, CYP3A4	2.01E-06
	hsa05204	Chemical carcinogenesis	CYP3A5, UGT2A2, EPHX1, CYP3A4	2.01E-06
	hsa00140	Steroid hormone biosynthesis	CYP3A5, UGT2A2, CYP3A4	0.00014
	hsa00830	Retinol metabolism	CYP3A5, UGT2A2, CYP3A4	0.00015
	hsa04976	Bile secretion	UGT2A2, EPHX1, CYP3A4	0.00029
	hsa00983	Drug metabolism - other enzymes	UGT2A2, CYP3A4	0.0194
2	hsa04141	Protein processing in endoplasmic reticulum	XBP1, HSP90B1, DNAJB11	0.00078
3	hsa00040	Pentose and glucuronate interconversions	CRYL1, AKR1A1	0.0062
4	hsa04151	PI3K-Akt signaling pathway	TSC2, FOXO3, MLST8	0.0075
	hsa04211	Longevity regulating pathway	TSC2, FOXO3	0.0202
	hsa04140	Autophagy - animal	TSC2, MLST8	0.0253
	hsa04150	mTOR signaling pathway	TSC2, MLST8	0.0253
	hsa04152	AMPK signaling pathway	TSC2, FOXO3	0.0253
	hsa04218	Cellular senescence	TSC2, FOXO3	0.0253
	hsa04714	Thermogenesis	TSC2, MLST8	0.0378
8	hsa04340	Hedgehog signaling pathway	SPOPL, SPOP	0.0058

This table details KEGG terms associated with various clusters, their descriptions, and lists proteins matched within the network alongside FDR values for a comprehensive enrichment analysis. The standard names of these genes or protein identifiers were unified by KEGG database

Similarly, 191 members of the greenyellow module were analyzed in the STRING database. After applying MCL clustering, 35 interacting members were categorized into 13 sub-networks (Fig. 4B). KEGG enrichment analysis revealed that Cluster 1 members were predominantly involved in pathways related to chemical carcinogenesis, steroid hormone biosynthesis, retinol metabolism, xenobiotic metabolism, and drug metabolism, underscoring their potential role in detoxification, drug metabolism, and hormone regulation (Table 2). Notably, the presence of P450 genes in both the green and greenyellow modules highlights their critical function in gene regulation during viral infections. Cluster 2 was associated with metabolic pathways, including amino sugar and nucleoside sugar metabolism, glycolipid biosynthesis, and glycosaminoglycan degradation, pointing to a role in glycan metabolism and cellular clearance. The enrichment of YARS1 and CARS1 in aminoacyl-tRNA biosynthesis in Cluster 4 indicates their importance in protein synthesis and metabolism. Meanwhile, the presence of CTSD, SMPD1, and CTSL in pathways like lysosome, neurosphingolipid signaling, autophagy, and apoptosis in Cluster 5 suggests their involvement in degradation processes, signaling, and programmed cell death. The involvement of CDIPT and MTMR14 in inositol phosphate metabolism and phosphatidylinositol signaling in Cluster 6 suggests a role in cell signaling and communication. The presence of SCARB2 and CD63 in the lysosomal pathway in Cluster 7 reflects their function in intracellular degradation and recycling. The recurring presence of SPOPL and SPOP in the Hedgehog signaling pathway in Cluster 11 emphasizes the pathway’s potential significance in the wasp’s response to PpNSRV-1 infection. Furthermore, the association of ADCY2 and RAPGEF3 with the Rap1 signaling pathway, cAMP signaling pathway, phosphatidylcholine signaling pathway, and adrenergic signaling in cardiomyocytes in Cluster 12 highlights their potential roles in cellular stress response and energy homeostasis.

**Table 2:** KEGG enrichment for the greenyellow module STRING functional network

Cluster	KEGG term	Term description	Matching proteins in network	FDR
1	hsa05204	Chemical carcinogenesis	CYP3A5, MGST1, ENSP00000480571, CYP3A4	4.22E-07
	hsa00140	Steroid hormone biosynthesis	CYP3A5, ENSP00000480571, CYP3A4	5.21E-05
	hsa00830	Retinol metabolism	CYP3A5, ENSP00000480571, CYP3A4	5.21E-05
	hsa00980	Metabolism of xenobiotics by cytochrome P450	CYP3A5, MGST1, CYP3A4	5.21E-05
	hsa00982	Drug metabolism - cytochrome P450	CYP3A5, MGST1, CYP3A4	5.21E-05
	hsa00983	Drug metabolism - other enzymes	MGST1, CYP3A4	0.0075
	hsa01100	Metabolic pathways	CYP3A5, MGST1, ENSP00000480571, CYP3A4	0.0075
	hsa04976	Bile secretion	ABCB1, CYP3A4	0.0086
2	hsa00520	Amino sugar and nucleotide sugar metabolism	HEXB, CHIT1, CHIA	1.94E-05
	hsa00604	Glycosphingolipid biosynthesis - ganglio series	HEXB, GLB1	0.00062
	hsa00511	Other glycan degradation	HEXB, GLB1	0.00066
	hsa00531	Glycosaminoglycan degradation	HEXB, GLB1	0.00066
	hsa01100	Metabolic pathways	HEXB, GLB1, CHIT1, CHIA	0.0019
	hsa04142	Lysosome	HEXB, GLB1	0.0137
4	hsa00970	Aminoacyl-tRNA biosynthesis	YARS1, CARS1	0.0054
5	hsa04142	Lysosome	CTSD, SMPD1, CTSL	9.00E-05
	hsa04071	Sphingolipid signaling pathway	CTSD, SMPD1	0.0179
	hsa04140	Autophagy - animal	CTSD, CTSL	0.0179
	hsa04210	Apoptosis	CTSD, CTSL	0.0179
6	hsa00562	Inositol phosphate metabolism	CDIPT, MTMR14	0.0047
	hsa04070	Phosphatidylinositol signaling system	CDIPT, MTMR14	0.0047
7	hsa04142	Lysosome	SCARB2, CD63	0.0139
11	hsa04340	Hedgehog signaling pathway	SPOPL, SPOP	0.002
12	hsa04015	Rap1 signaling pathway	ADCY2, RAPGEF3	0.0188
	hsa04024	cAMP signaling pathway	ADCY2, RAPGEF3	0.0188
	hsa04072	Phospholipase D signaling pathway	ADCY2, RAPGEF3	0.0188
	hsa04261	Adrenergic signaling in cardiomyocytes	ADCY2, RAPGEF3	0.0188

This table details KEGG terms associated with various clusters, their descriptions, and lists proteins matched within the network alongside FDR values for a comprehensive enrichment analysis. The standard names of these genes or protein identifiers were unified by KEGG database

### Pathways enriched in genes within the green module

**Fig. 5:**
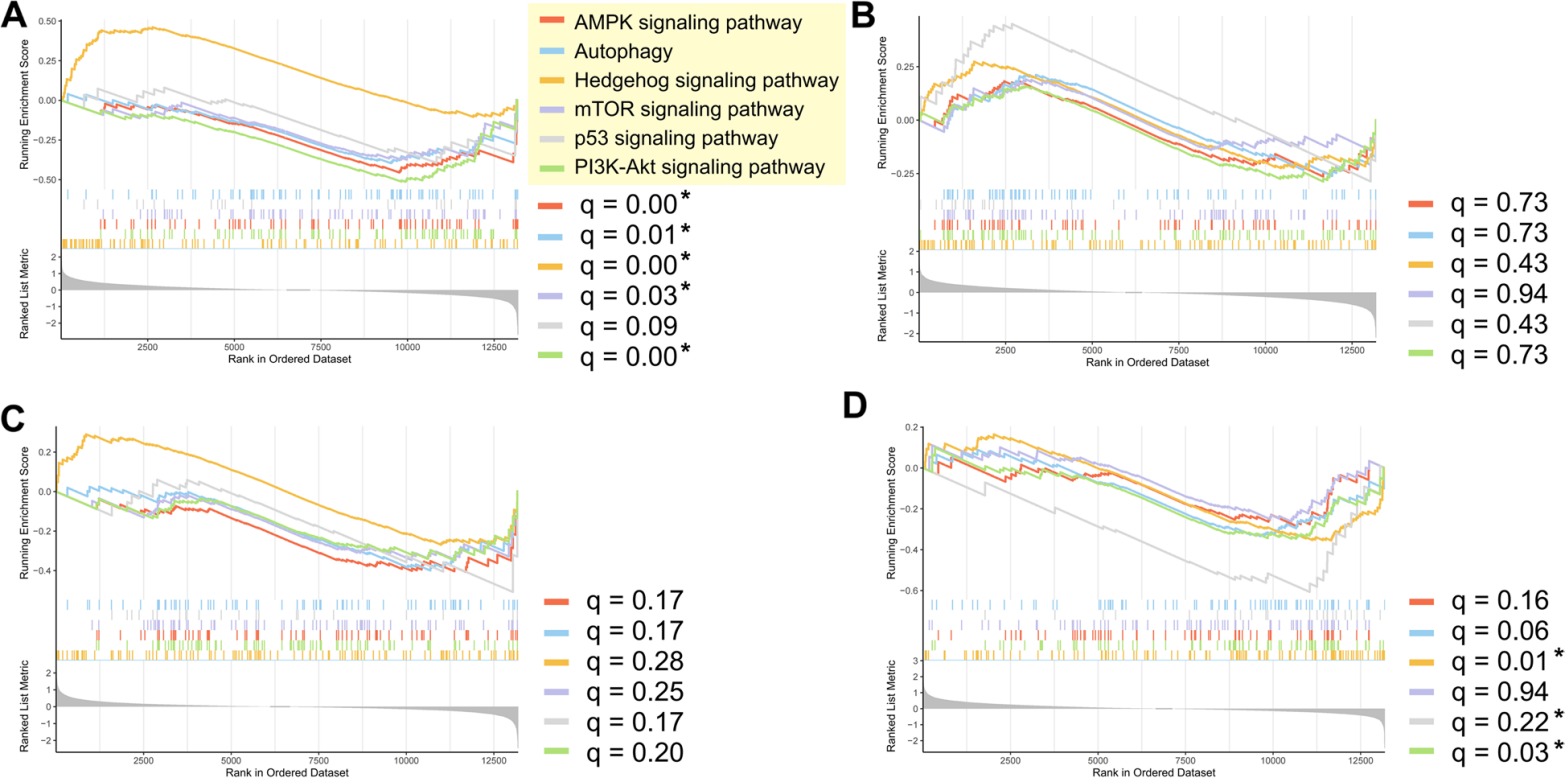
Gene set enrichment analysis. Enrichment results of autophagy, AMPK, Hedgehog, mTOR, p53, and PI3K-Akt signaling pathway genes in four age groups **A** 0 d; **B** 5 d; **C** 10 d; **D** 15 d

Gene regulatory network analysis identified several key signaling pathways and physiological processes potentially correlated with the extended lifespan in parasitoid wasps post PpNSRV-1 infection. Attention was directed towards the investigation of five critical signaling pathways: AMPK, Hedgehog, mTOR, p53, and PI3K-Akt, in addition to the autophagy process. All genes involved in these pathways were annotated and subjected to gene set enrichment analysis (GSEA). Notably, the Hedgehog signaling pathway exhibited significant dynamics, being highly activated at day 0, then showing a gradual decrease and marked inhibition in older age groups (Fig. 5A-D). The AMPK, PI3K-Akt signaling pathways, and autophagy consistently demonstrated an inhibited state (NES < 0) with significant levels (*q* < 0.05) at day 0. The mTOR and p53 signaling pathways, on the other hand, were activated at day 5 but did not reach significant levels (Table 3).

**Table 3:** NES for GSEA analysis of six signaling pathways in different age groups

**GeneSet**	**0 d**	**5 d**	**10 d**	**15 d**
PI3K-Akt signaling pathway	-1.927	-1.030	-1.208	-1.404
Hedgehog signaling pathway	1.765	1.169	1.085	-1.498
AMPK signaling pathway	-1.637	-0.917	-1.358	-1.107
Autophagy	-1.446	-0.918	-1.356	-1.305
mTOR signaling pathway	-1.364	0.762	-1.165	-1.017
p53 signaling pathway	-1.149	1.411	-1.384	-1.870

**Fig. 6:**
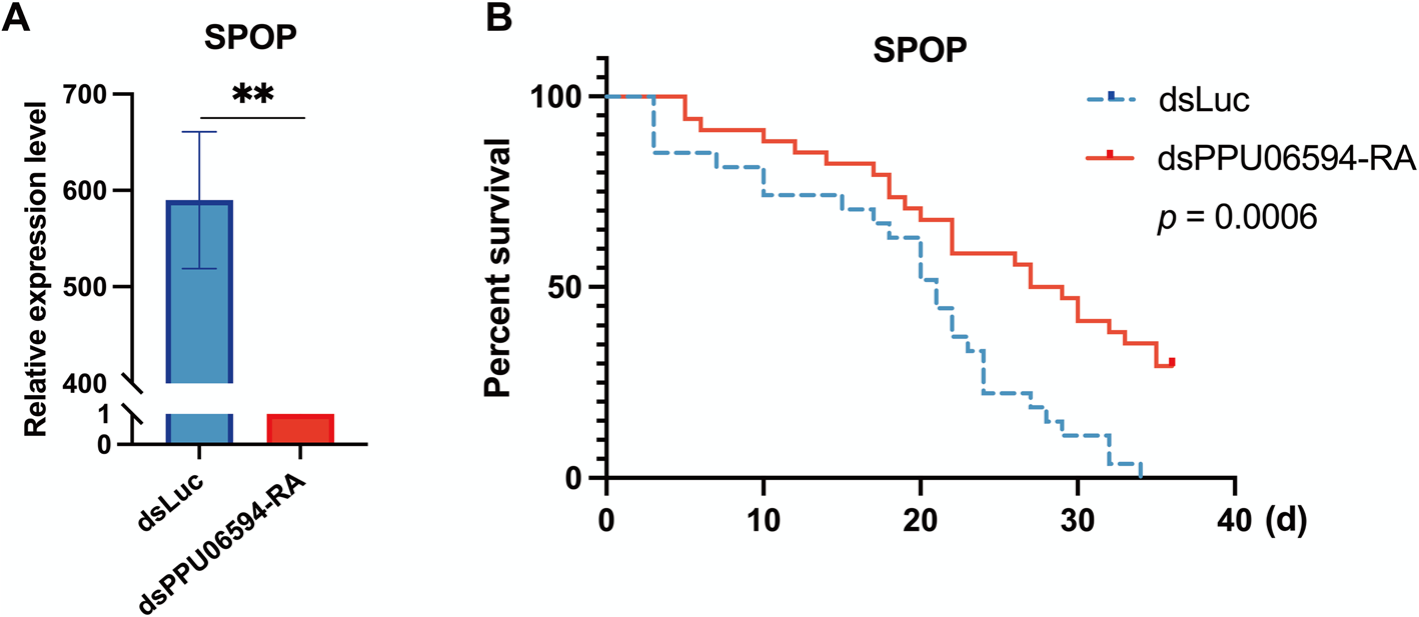
Effects of RNAi-mediated knock-down of SPOP gene on wasp survival. **A** Changes in the relative expression levels (REL) of *PPU06594-RA* after injection of dsPPU06594-RA; **B** Survival of wasps after injection of dsPPU06594-RA. ** means *p* < 0.01

The GSEA results, coupled with differential gene expression analysis, underscored the Hedgehog signaling pathway’s prominence, particularly noting that the SPOP gene *PPU06594-RA* on this pathway was significantly down-regulated across all age groups following PpNSRV-1 infection. Given the recognized importance of autophagy, AMPK, mTOR, p53, and PI3K-Akt pathways in lifespan regulation, alterations in their associated genes are anticipated to impact longevity. Consequently, we targeted the SPOP gene *PPU06594-RA* for RNA interference experiments to assess its influence on parasitoid wasp lifespan. Following dsPPU06594-RA injection, a significant reduction in *PPU06594-RA* expression was observed (*p* = 0.0012, Fig. 6A), and notably, wasps injected with dsPPU06594-RA exhibited a substantial lifespan increase compared to the dsLuc control group (Fig. 6B). This finding confirmed the role of the *SPOP* gene in modulating lifespan in parasitoid wasps.

## Discussion

This study, through comprehensive RNA-seq analysis, revealed potential transcriptional regulatory mechanisms contributing to the lifespan extension in parasitoid wasps after PpNSRV-1 virus infection, with an emphasis on the SPOP genes and the Hedgehog pathway.

The concurrent assessment of lifespan traits and transcriptome sequencing provided a solid foundation for correlating the observed lifespan extension with transcriptomic data. The clustering analysis of gene expression demonstrated a general trend of negative impact on gene expression due to viral infection. Among the DEGs, the SPOP genes and the P450 family members were particularly noteworthy. The P450s are known to be involved in a variety of biological processes, including drug metabolism, hormone biosynthesis, and toxin clearance [14]. Alterations in P450s expression may result from viral strategies aimed at evading host defenses, host metabolic reprogramming, or cellular stress response. The virus might suppress P450 expression to circumvent the host’s detoxification systems, thereby fostering a conducive environment for viral proliferation. Concurrently, the host may strategically down-regulate P450 enzymes to reallocate metabolic resources and bolster immune defense against the viral assault.

The application of the WGCNA workflow provided profound insights into the transcriptional changes induced by PpNSRV-1 virus infection in wasps. Two key gene modules, green and greenyellow, are significantly associated with virus-induced lifespan extension in wasps. The STRING functional network analysis of these modules revealed key genes within six pathways: AMPK, Hedgehog, mTOR, p53, and PI3K-Akt signaling pathways, as well as autophagy. These findings suggest a coordinated host response to the viral infection. These pathways are widely acknowledged for their influence on lifespan regulation across various biological models [15, 16]. The AMPK signaling pathway, known as a crucial cellular energy sensor, and the mTOR pathway, a key regulator of cell growth and metabolism, have both been shown to impact lifespan [17, 18]. For example, the average lifespan of mice was found to be increased by 20% through decreasing mTOR expression in mice [19]. Similarly, the p53 protein, a guardian of genomic stability and a suppressor of tumorigenesis, contributes to cellular senescence and apoptosis, influencing tissue health and longevity [20]. The PI3K-Akt signaling pathway, integral for cell growth, differentiation, and survival, has been associated with viral infection, carcinogenesis, and lifespan regulation [21]. Moreover, autophagy, an essential intracellular recycling mechanism, maintains cellular homeostasis and tissue health, with its proper functioning recognized as crucial for lifespan [22]. Inhibition of these pathways at an early adult stage may have contributed to the extended lifespan of wasps.

Both the green and greenyellow modules underscored the critical role of SPOP genes and the Hedgehog pathway. During the early adult stage of wasps, the Hedgehog pathway experienced an up-regulation, gradually transitioning to down-regulation as the wasps aged, with significant down-regulation observed in older wasps. This temporal modulation, mirroring the virus replication timeline, suggests a potential correlation between the regulation of the Hedgehog pathway and extended lifespan in adult wasps. The Hedgehog signaling pathway is a conserved network that regulates numerous developmental and physiological processes in both invertebrates and vertebrates [23, 24]. It has been implicated in various disorders when dysregulated [25]. Over-expression of the Hedgehog pathway prolongs the lifespan of *D. melanogaster* [26]. The interaction of Hh ligands with the Patched receptor initiates a cascade of events, culminating in the nuclear translocation of Gli transcription factors and the regulation of genes like *BMP4*, *SPOP*, and *EN*. *SPOP* encodes a substrate-binding protein for the Cullin3-based E3 ubiquitin ligase complex and is involved in the regulation of various cellular processes, including protein homeostasis, cell cycle progression, and DNA damage response [27]. The SPOP gene, serving as a regulatory hub, plays a dual role in promoting or inhibiting tumorigenesis through the ubiquitination and degradation of multiple substrates [28]. RNAi experiments targeting the consistently down-regulated SPOP gene *PPU06594-RA* in early adult wasps led to a marked increase in survival rates, underscoring the role of SPOP genes in lifespan regulation. This suggests that the down-regulation of SPOP genes following viral infection may contribute to the observed lifespan extension in host wasps.

## Materials and methods

### Construction of near-isogenic lines of parasitoids with and without PpNSRV-1

The laboratory *P. puparum* colonies, with and without PpNSRV-1, were reared in an artificial climate incubator with standard conditions (25 ± 1 °C, 60 ± 5% relative humidity, and a 16: 8 light-dark photoperiod) [29]. To establish the near-isogenic line, the ovaries of 30 female wasps from the PpNSRV-1(+) population were homogenized and centrifuged to obtain the crude virus solution. The crude virus was microinjected into PpNSRV-1(-) wasps for infection, and then self-crossed for several generations. Two distinct groups were established from field-collected parasitoid wasp populations based on their PpNSRV-1 viral status. The primary genetic background for this experiment was the PpNSRV-1-free population. To ensure genetic homogeneity, we introduced PpNSRV-1 into the virus-free population, and systematically purified the infected line over multiple generations. The sequenced wasps included PpNSRV-1-infected (I(+)) and non-infected (I(-)) individuals.

### RNA-seq analyses

Samples from 120 wasps (5 individuals per genetic line per treatment) were collected at 0, 5, 10 and 15 days of age for RNA isolation (Fig. 1A). Female wasps carrying PpNSRV-1 were designated as I(+), and those without PpNSRV-1 as I(-). Total RNA was extracted from each sample using Trizol reagent (Invitrogen, Carlsbad, CA, USA) following the manufacturer’s instructions. The quality and quantity of the extracted RNA were assessed using a NanoDrop spectrophotometer (Thermo Fisher Scientific, Waltham, MA, USA). For library preparation, the Illumina TruSeqTM RNA Sample Prep Kit method (Illumina, NEB, United States) was employed. The final cDNA libraries were quantified using a Qubit fluorometer (Thermo Fisher Scientific, Waltham, MA, USA) and validated for size distribution using the Agilent Bioanalyzer High Sensitivity DNA Assay. Sequencing of the cDNA libraries was performed using the Illumina HiSeq platform to generate paired-end reads. The raw sequencing data obtained from the HiSeq runs were subjected to quality control assessment using FastQC software. Adapter sequences and low-quality bases were trimmed using Sickle software. The trimmed reads were aligned to the reference genome using bowtie2 (version 2.4.4) with default parameters. RSEM (version 1.3.0) was used to quantify gene level and conveyed as fragments per kilobase of transcript per million (FPKM) [30]. An R package DESeq2 (version 1.22.2) was used for differential expression analysis [31]. The sequence data have been submitted through the Sequence Read Archive (https://www.ncbi.nih.gov/sra/) under accession number SUB13085452. Two major factors were included for the RNA-seq analyses: PpNSRV-1 (with, without) and age (0 d, 5 d, 10 d, 15 d). Gene read counts of I(+) were compared to I(-) at the same age. The criteria of DEGs were FDR < 0.05 and log_2_|Fold Change|> 1. Enrichment analyses of Gene ontology (GO) and KEGG pathway were performed and plotted using OmicShare tools (http://www.omicshare.com/tools). GO terms and KEGG pathways with *q* < 0.05 were considered as statistically enriched.

### Weighed gene co-expression network analysis

Weighted gene co-expression network analysis was constructed using the R package WGCNA v1.66 [32]. Genes with low expression (FPKM = 0) were filtered out. The network was constructed using the following parameters: unsigned network type, soft power of 2, dynamic tree cut for module identification, minimum module size of 30, and merge modules with high similarity of 0.2. Default values were used unless specified. The correlation between gene modules and the traits, including PpNSRV-1 and age, was assessed. First, PCA was performed on the genes within a module, with the PC1 used to represent the ME, which reflects the traits of this module. The correlation coefficient between ME and traits was calculated and visualized as a heat map. GS was determined for the traits of interest, including PpNSRV-1 infection and age, and MM were calculated. Genes within the target modules were further analyzed with STRING database [33].

### Gene set enrichment analysis

Gene Set Enrichment Analysis (GSEA) was used to study gene expression patterns and their association with biological functions in transcriptome data [34]. First, all genes ranked at each age based on their FPKM values. Next, six signaling pathway gene sets were selected for analysis, and all genes in the target pathway were annotated in the *P. puparum* genome to construct a gene set containing the names of all genes in the target pathway.

Using GSEA software, the Enrichment Score (ES) of each gene set was calculated based on the ranking of the genes and the membership of the selected gene sets. The ES reflects the distribution of the gene set across the entire ranked list, and a high ES indicates that the gene set is enriched in the top-ranked genes. To assess the significance of the Enrichment Score, multiple random sampling was performed using a ranking-based approach to generate the null distribution and calculate the Normalized Enrichment Score (NES). By comparing the actual score and the null distribution, the hypothesis testing *p*-value and False Discovery Rate (FDR) were calculated for each gene set. Based on the NES and FDR, gene sets that were significantly enriched at each sampling time point were identified and further analyzed.

### Preparation of dsRNA and RNAi assays

To investigate the functional significance of the SPOP gene on *P. puparum*, RNAi knockdown of the genes was utilized to reduce the abundance of the SPOP gene, and then the effect of these knockdowns on wasp survival was evaluated. We used two different double-stranded RNA (dsRNA) fragments to exclude off-target effects. After detecting the RNAi efficiency by pre-experiment, the pair of primers with the best efficiency was selected (dsPPU06594-RA-F1/dsPPU06594-RA-R1).

The SPOP gene-specific primers and primers targeting luciferase (Luc; negative control) were designed with added T7 promoter adaptors (Table S3). All amplified PCR products were cloned into pCE2 TA/Blunt-Zero vector (Vazyme, Nanjing, China) and sequenced. The correct PCR products were used as templates for dsRNA synthesis with the T7 High Yield RNA Transcription Kit (Vazyme, Nanjing, China), according to the manufacturer’s instructions. Synthesized dsRNA was quantified using a NanoDrop 2000 Spectrophotometer (Thermo Scientific, Wilmington, DE) at 260 nm.

30 nL of dsRNA (3 × 10^3^ ng/μL) was injected into each yellow wasp pupa using Drummond Nanoject II™ Auto-Nanoliter Injector (Drummond Scientific Company, Broomall, United States). The expression level of the gene in the wasps emerging from injected wasp pupae was quantified by qPCR. Then, emerging wasps were all collected and placed in a suitable environment to measure longevity.

## Supplementary Information

Supplementary Material 1: Table S1. Clean reads data of RNA-seq. Table S2. KEGG pathway enrichment analysis of gene clusters identified by mfuzz. Table S3. All primers used in this study. Fig. S1. Results of PCA analysis of samples. Fig. S2. Filtering of optimal soft thresholds.

## Data Availability

The authors confirm that the data supporting the findings of this study are available within the article and its supplementary materials.
