# Supplementary material for: Transcriptomic alterations in host parasitoid wasps resulting in extended lifespan due to PpNSRV-1 infection
Source: Crop Health. 2024 Jun 5;2(1):9. doi: 10.1007/s44297-024-00029-w (PMC12825976; doi:10.1007/s44297-024-00029-w)
Supplement: Supplementary file 1 — Supplementary Material 1: Table S1. Clean reads data of RNA-seq. Table S2. KEGG pathway enrichment analysis of gene clusters identified by mfuzz. Table S3. All primers used in this study. Fig. S1. Results of PCA analysis of samples. Fig. S2. Filtering of optimal soft thresholds. [file 44297_2024_29_MOESM1_ESM.docx]

**Supplementary Information**

**Transcriptomic alterations in host parasitoid wasps resulting in extended lifespan due to PpNSRV-1 infection**

**Cheng Xue^1,2^, Fei Wang^1^, Qi Fang^1^, Shijiao Xiong^1,3*^, Gongyin Ye^1^**

**^1^State Key Laboratory of Rice Biology and Breeding, Institute of Insect Sciences, Zhejiang University, Hangzhou 310058, China**

**^2^Hainan Institute of Zhejiang University, Sanya 572025, China**

**^3^Xianghu Laboratory, Hangzhou 311231, China**

***Corresponding to: Shijiao Xiong, xiongshijiao@zju.edu.cn**

**Figures**


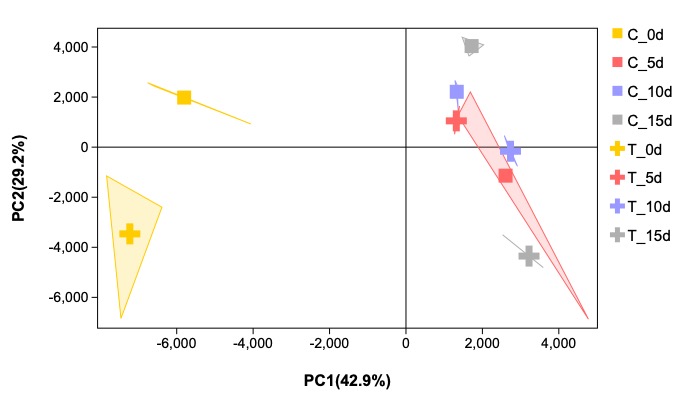


**Fig. S1. Results of PCA analysis of samples.** The squares represent the virus-uninfected control group and the crosses represent the virus-infected treatment group. Three biological replicates of each treatment group were plotted by taking the center of mass, and the three vertices of the connecting line represent each biological replicate.


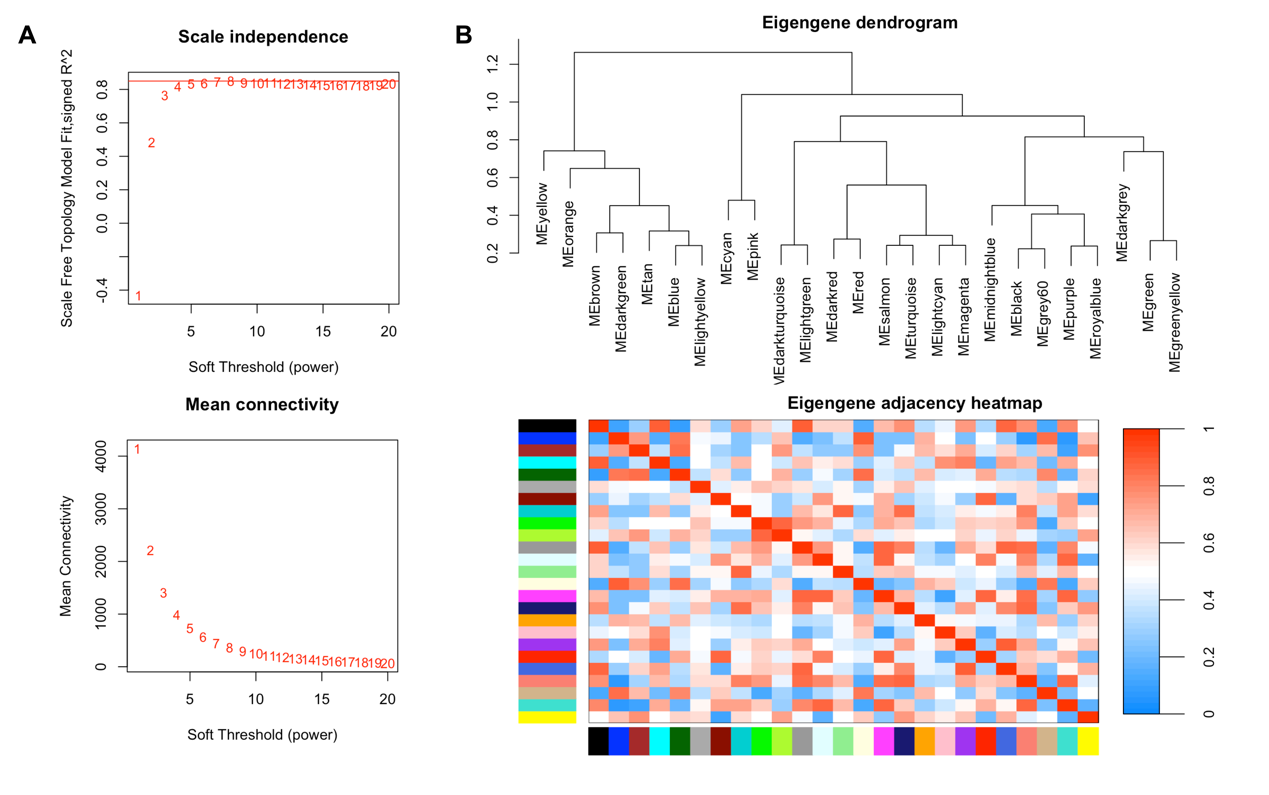


**Fig. S2. Filtering of optimal soft thresholds.** (A) Network scale for different soft threshold values. (B) Mean connectivity for different soft threshold values.

**Tables**

**Table S1. Clean reads data of RNA-seq**

| **Sample ID** | **Trait** | **Reads** | **Bases（bp）** | **Error%** | **Q30%** | **GC%** |
| --- | --- | --- | --- | --- | --- | --- |
| 0d-Fm(-)-1 | PpNSRV-1(-) | 30608569 | 4413357555 | 0.0125 | 94.61 | 44.45 |
| 0d-Fm(-)-2 | PpNSRV-1(-) | 30811050 | 4444960266 | 0.0125 | 94.57 | 44.57 |
| 0d-Fm(-)-3 | PpNSRV-1(-) | 25150505 | 3611544246 | 0.0127 | 94.42 | 44.38 |
| 5d-Fm(-)-1 | PpNSRV-1(-) | 30600584 | 4417935793 | 0.0122 | 95.14 | 44.91 |
| 5d-Fm(-)-2 | PpNSRV-1(-) | 32023961 | 4623711643 | 0.0121 | 95.21 | 44.53 |
| 5d-Fm(-)-3 | PpNSRV-1(-) | 46424212 | 6749465469 | 0.0122 | 95.04 | 45.83 |
| 10d-Fm(-)-1 | PpNSRV-1(-) | 35042336 | 5079836920 | 0.0121 | 95.15 | 43.66 |
| 10d-Fm(-)-2 | PpNSRV-1(-) | 42878624 | 6219628312 | 0.012 | 95.33 | 43.49 |
| 10d-Fm(-)-3 | PpNSRV-1(-) | 37166733 | 5385167142 | 0.012 | 95.31 | 43.39 |
| 15d-Fm(-)-1 | PpNSRV-1(-) | 41741043 | 6044064810 | 0.0123 | 94.92 | 44.24 |
| 15d-Fm(-)-2 | PpNSRV-1(-) | 36171366 | 5252292049 | 0.012 | 95.24 | 44.37 |
| 15d-Fm(-)-3 | PpNSRV-1(-) | 21486758 | 3100647657 | 0.0124 | 94.84 | 44.28 |
| 0d-Fm(+)-1 | PpNSRV-1(+) | 26298715 | 3772752757 | 0.0126 | 94.43 | 44.49 |
| 0d-Fm(+)-2 | PpNSRV-1(+) | 33405204 | 4803081616 | 0.0125 | 94.67 | 44.01 |
| 0d-Fm(+)-3 | PpNSRV-1(+) | 25847867 | 3708284152 | 0.0126 | 94.51 | 44.65 |
| 5d-Fm(+)-1 | PpNSRV-1(+) | 31993322 | 4621938444 | 0.0125 | 94.59 | 44.48 |
| 5d-Fm(+)-2 | PpNSRV-1(+) | 31151644 | 4478457002 | 0.0127 | 94.35 | 44.36 |
| 5d-Fm(+)-3 | PpNSRV-1(+) | 35123679 | 5087512808 | 0.0122 | 94.95 | 44.75 |
| 10d-Fm(+)-1 | PpNSRV-1(+) | 29922354 | 4319261895 | 0.0122 | 95.14 | 43.53 |
| 10d-Fm(+)-2 | PpNSRV-1(+) | 31288108 | 4526567791 | 0.0121 | 95.24 | 44.43 |
| 10d-Fm(+)-3 | PpNSRV-1(+) | 36170837 | 5253423120 | 0.012 | 95.27 | 43.82 |
| 15d-Fm(+)-1 | PpNSRV-1(+) | 33267895 | 4815958697 | 0.0122 | 95.03 | 44.19 |
| 15d-Fm(+)-2 | PpNSRV-1(+) | 28607469 | 4105780250 | 0.0124 | 94.79 | 43.83 |
| 15d-Fm(+)-3 | PpNSRV-1(+) | 50675350 | 7405754092 | 0.0124 | 94.85 | 45.33 |

**Table S2. KEGG pathway enrichment analysis of gene clusters identified by mfuzz**

| **Pathway** | ***p* value** | ***q* value** | **Pathway ID** |
| --- | --- | --- | --- |
| **Cluster 3** |  |  |  |
| Alzheimer disease | 0.000 | 0.000 | ko05010 |
| Parkinson disease | 0.000 | 0.000 | ko05012 |
| Prion disease | 0.000 | 0.000 | ko05020 |
| Pathways of neurodegeneration - multiple diseases | 0.000 | 0.000 | ko05022 |
| Huntington disease | 0.000 | 0.000 | ko05016 |
| Diabetic cardiomyopathy | 0.000 | 0.000 | ko05415 |
| Amyotrophic lateral sclerosis | 0.000 | 0.000 | ko05014 |
| Oxidative phosphorylation | 0.000 | 0.000 | ko00190 |
| Thermogenesis | 0.000 | 0.000 | ko04714 |
| Chemical carcinogenesis - reactive oxygen species | 0.000 | 0.000 | ko05208 |
| Spinocerebellar ataxia | 0.000 | 0.000 | ko05017 |
| Non-alcoholic fatty liver disease | 0.000 | 0.000 | ko04932 |
| Neuroactive ligand-receptor interaction | 0.000 | 0.003 | ko04080 |
| Proteasome | 0.000 | 0.009 | ko03050 |
| Peroxisome | 0.000 | 0.009 | ko04146 |
| Retrograde endocannabinoid signaling | 0.001 | 0.020 | ko04723 |
| Phototransduction - fly | 0.001 | 0.023 | ko04745 |
| Circadian entrainment | 0.001 | 0.026 | ko04713 |
| Viral myocarditis | 0.002 | 0.027 | ko05416 |
| **Cluster 4** |  |  |  |
| Cell cycle | 0.000 | 0.001 | ko04110 |
| Cell cycle - yeast | 0.000 | 0.001 | ko04111 |
| Nucleotide excision repair | 0.000 | 0.001 | ko03420 |
| Ribosome biogenesis in eukaryotes | 0.000 | 0.003 | ko03008 |
| Fanconi anemia pathway | 0.000 | 0.007 | ko03460 |
| Mannose type O-glycan biosynthesis | 0.001 | 0.020 | ko00515 |
| Meiosis - yeast | 0.001 | 0.020 | ko04113 |
| Spliceosome | 0.001 | 0.038 | ko03040 |
| DNA replication | 0.001 | 0.038 | ko03030 |
| **Cluster 5** |  |  |  |
| Axon guidance | 0.000 | 0.000 | ko04360 |
| MicroRNAs in cancer | 0.000 | 0.000 | ko05206 |
| Cellular senescence | 0.000 | 0.000 | ko04218 |
| PI3K-Akt signaling pathway | 0.000 | 0.000 | ko04151 |
| Human T-cell leukemia virus 1 infection | 0.000 | 0.000 | ko05166 |
| Autophagy - animal | 0.000 | 0.000 | ko04140 |
| Human cytomegalovirus infection | 0.000 | 0.000 | ko05163 |
| FoxO signaling pathway | 0.000 | 0.000 | ko04068 |
| MAPK signaling pathway | 0.000 | 0.000 | ko04010 |
| Pathways in cancer | 0.000 | 0.000 | ko05200 |
| Endocrine resistance | 0.000 | 0.001 | ko01522 |
| ErbB signaling pathway | 0.000 | 0.001 | ko04012 |
| Chronic myeloid leukemia | 0.000 | 0.001 | ko05220 |
| Breast cancer | 0.000 | 0.001 | ko05224 |
| VEGF signaling pathway | 0.000 | 0.001 | ko04370 |
| Signaling pathways regulating pluripotency of stem cells | 0.000 | 0.001 | ko04550 |
| mTOR signaling pathway | 0.000 | 0.001 | ko04150 |
| Cushing syndrome | 0.000 | 0.001 | ko04934 |
| Human papillomavirus infection | 0.000 | 0.001 | ko05165 |
| Hepatocellular carcinoma | 0.000 | 0.001 | ko05225 |
| Phospholipase D signaling pathway | 0.000 | 0.001 | ko04072 |
| Lipid and atherosclerosis | 0.000 | 0.002 | ko05417 |
| T cell receptor signaling pathway | 0.000 | 0.002 | ko04660 |
| Chemokine signaling pathway | 0.000 | 0.003 | ko04062 |
| Fc epsilon RI signaling pathway | 0.000 | 0.003 | ko04664 |
| p53 signaling pathway | 0.000 | 0.003 | ko04115 |
| Osteoclast differentiation | 0.000 | 0.003 | ko04380 |
| Thyroid hormone signaling pathway | 0.000 | 0.003 | ko04919 |
| Progesterone-mediated oocyte maturation | 0.000 | 0.003 | ko04914 |
| Neurotrophin signaling pathway | 0.000 | 0.003 | ko04722 |
| Prostate cancer | 0.000 | 0.003 | ko05215 |
| GnRH signaling pathway | 0.000 | 0.003 | ko04912 |
| Hepatitis B | 0.000 | 0.003 | ko05161 |
| Longevity regulating pathway | 0.000 | 0.004 | ko04211 |
| B cell receptor signaling pathway | 0.001 | 0.004 | ko04662 |
| Melanogenesis | 0.001 | 0.004 | ko04916 |
| Renal cell carcinoma | 0.001 | 0.004 | ko05211 |
| Gastric cancer | 0.001 | 0.004 | ko05226 |
| Melanoma | 0.001 | 0.005 | ko05218 |
| Cell cycle | 0.001 | 0.005 | ko04110 |
| Human immunodeficiency virus 1 infection | 0.001 | 0.005 | ko05170 |
| Growth hormone synthesis, secretion and action | 0.001 | 0.005 | ko04935 |
| Non-small cell lung cancer | 0.001 | 0.005 | ko05223 |
| Wnt signaling pathway | 0.001 | 0.006 | ko04310 |
| Natural killer cell mediated cytotoxicity | 0.001 | 0.006 | ko04650 |
| Kaposi sarcoma-associated herpesvirus infection | 0.001 | 0.006 | ko05167 |
| Proteoglycans in cancer | 0.001 | 0.006 | ko05205 |
| Viral carcinogenesis | 0.001 | 0.006 | ko05203 |
| MAPK signaling pathway - fly | 0.001 | 0.006 | ko04013 |
| Sphingolipid signaling pathway | 0.001 | 0.006 | ko04071 |
| AMPK signaling pathway | 0.001 | 0.006 | ko04152 |
| Pancreatic cancer | 0.002 | 0.010 | ko05212 |
| Bladder cancer | 0.002 | 0.011 | ko05219 |
| Endocytosis | 0.002 | 0.013 | ko04144 |
| Adherens junction | 0.003 | 0.014 | ko04520 |
| Relaxin signaling pathway | 0.003 | 0.015 | ko04926 |
| Parathyroid hormone synthesis, secretion and action | 0.003 | 0.015 | ko04928 |
| Acute myeloid leukemia | 0.003 | 0.016 | ko05221 |
| Oxytocin signaling pathway | 0.003 | 0.016 | ko04921 |
| Tight junction | 0.004 | 0.017 | ko04530 |
| C-type lectin receptor signaling pathway | 0.004 | 0.017 | ko04625 |
| Glioma | 0.004 | 0.017 | ko05214 |
| Axon regeneration | 0.004 | 0.017 | ko04361 |
| Choline metabolism in cancer | 0.004 | 0.020 | ko05231 |
| Chemical carcinogenesis - receptor activation | 0.005 | 0.022 | ko05207 |
| Estrogen signaling pathway | 0.005 | 0.023 | ko04915 |
| Neutrophil extracellular trap formation | 0.005 | 0.023 | ko04613 |
| Oocyte meiosis | 0.006 | 0.024 | ko04114 |
| Toll-like receptor signaling pathway | 0.006 | 0.025 | ko04620 |
| Glutamatergic synapse | 0.006 | 0.025 | ko04724 |
| AGE-RAGE signaling pathway in diabetic complications | 0.006 | 0.025 | ko04933 |
| Fanconi anemia pathway | 0.007 | 0.027 | ko03460 |
| Gap junction | 0.007 | 0.027 | ko04540 |
| Herpes simplex virus 1 infection | 0.007 | 0.028 | ko05168 |
| JAK-STAT signaling pathway | 0.008 | 0.029 | ko04630 |
| Endometrial cancer | 0.008 | 0.029 | ko05213 |
| PD-L1 expression and PD-1 checkpoint pathway in cancer | 0.008 | 0.029 | ko05235 |
| Glucagon signaling pathway | 0.008 | 0.029 | ko04922 |
| Notch signaling pathway | 0.009 | 0.033 | ko04330 |
| Pathogenic Escherichia coli infection | 0.010 | 0.035 | ko05130 |
| EGFR tyrosine kinase inhibitor resistance | 0.010 | 0.035 | ko01521 |
| Th17 cell differentiation | 0.010 | 0.036 | ko04659 |
| Insulin signaling pathway | 0.010 | 0.036 | ko04910 |
| Renin secretion | 0.012 | 0.042 | ko04924 |
| **Cluster 6** |  |  |  |
| Hippo signaling pathway | 0.000 | 0.003 | ko04390 |
| Hippo signaling pathway - fly | 0.000 | 0.027 | ko04391 |
| Ubiquitin mediated proteolysis | 0.000 | 0.027 | ko04120 |
| Protein processing in endoplasmic reticulum | 0.000 | 0.030 | ko04141 |
| Apoptosis - fly | 0.001 | 0.045 | ko04214 |
| Shigellosis | 0.001 | 0.049 | ko05131 |
| **Cluster 7** |  |  |  |
| Nucleocytoplasmic transport | 0.000 | 0.008 | ko03013 |
| Systemic lupus erythematosus | 0.000 | 0.008 | ko05322 |
| Cell cycle - yeast | 0.000 | 0.008 | ko04111 |
| Spliceosome | 0.000 | 0.025 | ko03040 |
| Cluster 9 |  |  |  |
| Coronavirus disease - COVID-19 | 0.000 | 0.000 | ko05171 |
| Ribosome | 0.000 | 0.000 | ko03010 |
| Parkinson disease | 0.000 | 0.020 | ko05012 |
| Prion disease | 0.000 | 0.028 | ko05020 |
| **Cluster 10** |  |  |  |
| GABAergic synapse | 0.000 | 0.000 | ko04727 |
| Axon regeneration | 0.000 | 0.001 | ko04361 |
| Insulin resistance | 0.000 | 0.001 | ko04931 |
| Morphine addiction | 0.000 | 0.001 | ko05032 |
| AGE-RAGE signaling pathway in diabetic complications | 0.000 | 0.002 | ko04933 |
| Insulin secretion | 0.000 | 0.004 | ko04911 |
| Proteoglycans in cancer | 0.000 | 0.004 | ko05205 |
| Synaptic vesicle cycle | 0.000 | 0.007 | ko04721 |
| Relaxin signaling pathway | 0.000 | 0.010 | ko04926 |
| Adrenergic signaling in cardiomyocytes | 0.000 | 0.010 | ko04261 |
| Retrograde endocannabinoid signaling | 0.000 | 0.010 | ko04723 |
| Leukocyte transendothelial migration | 0.000 | 0.010 | ko04670 |
| Taste transduction | 0.000 | 0.012 | ko04742 |
| Aldosterone synthesis and secretion | 0.001 | 0.014 | ko04925 |
| Platelet activation | 0.001 | 0.015 | ko04611 |
| Inflammatory mediator regulation of TRP channels | 0.001 | 0.015 | ko04750 |
| Cholinergic synapse | 0.001 | 0.018 | ko04725 |
| Growth hormone synthesis, secretion and action | 0.001 | 0.018 | ko04935 |
| Pathways in cancer | 0.001 | 0.022 | ko05200 |
| Thyroid hormone synthesis | 0.001 | 0.024 | ko04918 |
| Purine metabolism | 0.002 | 0.027 | ko00230 |
| Adipocytokine signaling pathway | 0.002 | 0.027 | ko04920 |
| Focal adhesion | 0.002 | 0.027 | ko04510 |
| Citrate cycle (TCA cycle) | 0.002 | 0.027 | ko00020 |
| Chemokine signaling pathway | 0.002 | 0.027 | ko04062 |
| Serotonergic synapse | 0.002 | 0.031 | ko04726 |
| Human cytomegalovirus infection | 0.003 | 0.038 | ko05163 |
| Nicotine addiction | 0.003 | 0.040 | ko05033 |
| Proximal tubule bicarbonate reclamation | 0.004 | 0.041 | ko04964 |
| Amoebiasis | 0.004 | 0.041 | ko05146 |
| Salivary secretion | 0.005 | 0.048 | ko04970 |

**Table S3. All primers used in this study**

| **Primer name** | **Sequence (5’ to 3’)** | **Usage** |
| --- | --- | --- |
| PPU06594-RA-F1 | TGGTATCTTTCTATGCTTCC | Cloning |
| PPU06594-RA-R1 | TGCTTCTGAGTGTTTCGTC |  |
| PPU06594-RA-F2 | TGGTATCTTTCTATGCTTCC |  |
| PPU06594-RA-R2 | CATTTCGCTTTCAGTCCTT |  |
| dsPPU06594-RA-F1 | TAATACGACTCACTATAGGGAGATGGTATCTTTCTATGCTTCC | dsRNA |
| dsPPU06594-RA-R1 | TAATACGACTCACTATAGGGAGATGCTTCTGAGTGTTTCGTC |  |
| dsPPU06594-RA-F2 | TAATACGACTCACTATAGGGAGATGGTATCTTTCTATGCTTCC |  |
| dsPPU06594-RA-R2 | TAATACGACTCACTATAGGGAGACATTTCGCTTTCAGTCCTT |  |
| dsLuc-F | TAATACGACTCACTATAGGGAGATGGAATGTTTACTACACTCG |  |
| dsLuc-R | TAATACGACTCACTATAGGGAGACATAATCATAGGACCTCTCA |  |
| qPPU06594-RA-F1 | AGTAAACGGTGTTATTCG | qPCR |
| qPPU06594-RA-R1 | AACTTTCATTGCCAGGT |  |
| qPPU06594-RA-F2 | ACACGGATAAAGTTGACGGCG |  |
| qPPU06594-RA-R2 | CAGTGAAGGGATGTTTGGCTA |  |
